# Fast Disintegrating Quercetin-Loaded Drug Delivery Systems Fabricated Using Coaxial Electrospinning

**DOI:** 10.3390/ijms141121647

**Published:** 2013-10-31

**Authors:** Xiao-Yan Li, Yan-Chun Li, Deng-Guang Yu, Yao-Zu Liao, Xia Wang

**Affiliations:** 1 School of Materials Science & Engineering, University of Shanghai for Science and Technology, Shanghai 200093, China; E-Mails: lixiaoyan@usst.edu.cn (X.-Y.L.); longphoon@163.com (Y.-C.L.); 2 School of Chemistry, University of Bristol, Bristol, England BS8 1TS, UK; E-Mail: yaozu.liao@bristol.ac.uk

**Keywords:** nanocomposites, core-sheath nanofibres, coaxial electrospinning, fast disintegrating, quercetin

## Abstract

The objective of this study is to develop a structural nanocomposite of multiple components in the form of core-sheath nanofibres using coaxial electrospinning for the fast dissolving of a poorly water-soluble drug quercetin. Under the selected conditions, core-sheath nanofibres with quercetin and sodium dodecyl sulphate (SDS) distributed in the core and sheath part of nanofibres, respectively, were successfully generated, and the drug content in the nanofibres was able to be controlled simply through manipulating the core fluid flow rates. Field emission scanning electron microscope (FESEM) images demonstrated that the nanofibres prepared from the single sheath fluid and double core/sheath fluids (with core-to-sheath flow rate ratios of 0.4 and 0.7) have linear morphology with a uniform structure and smooth surface. The TEM images clearly demonstrated the core-sheath structures of the produced nanocomposites. Differential scanning calorimetry (DSC) and X-ray diffraction (XRD) results verified that quercetin and SDS were well distributed in the polyvinylpyrrolidone (PVP) matrix in an amorphous state, due to the favourite second-order interactions. *In vitro* dissolution studies showed that the core-sheath composite nanofibre mats could disintegrate rapidly to release quercetin within 1 min. The study reported here provides an example of the systematic design, preparation, characterization and application of a new type of structural nanocomposite as a fast-disintegrating drug delivery system.

## 1. Introduction

The solubility behaviour of poorly water-soluble drugs is one of the most challenging aspects of formulation development in pharmaceutics [1]. Nanosizing strategies can be used to enhance the dissolution and oral availability of numerous poorly soluble drugs by enlarging the surface area of the drug powder and/or changing the crystalline form [2,3]. Among different nanoproducts (such as nanoparticles, nanocrystalline particles, nanosuspensions of pure drugs, solid lipid nanoparticles, microemulsions, micelles and nanoencapsulations [4]), electrospun composite nanofibres have shown their potential in this field most recently [5–7].

Electrospinning is a simple and straightforward process for generating nanofibres. The popularity of this system is due to its easy implementation, capability of treating a variety of materials, convenience in obtaining composites of multiple components and a wide variety of potential applications of the resultant nanofibres [8–11]. Electrospinning shares characteristics of both electrospraying and conventional solution dry spinning of fibres and is inherently an appropriate method for preparing nanocomposites [12,13]. The fast drying electrospinning process is able to ‘freeze’ the drug molecules randomly in the solid polymer fibre matrix, into a state comparable to a liquid form. This is very useful to prevent phase separation, e.g., re-crystallization of either drug or matrix, during removal of the solvents [14].

Fast-dissolving delivery systems (FDDS) address the needs of populations requiring special attention, such as paediatric and geriatric patients. Difficulty in swallowing medicines is often encountered by these patients, leading to non-compliance with medication [15]. FDDS offer additional advantages, such as more rapid drug absorption, extension of the patent life of existing drugs, elimination of the need for water and increased ease of taking medicines while traveling and for patients with restricted water intake [16]. The demand for FDDS has continuously increased. Oral FDDS include fast-disintegrating tablets, fast-disintegrating capsules, fast-dissolving strips and fast-dissolving mucoadhesive microparticulates and membranes [5]. As an emerging novel dosage form, oral fast-dissolving membranes (FDMs), which can dissolve readily on the tongue to deliver drugs to a patient and replace the use of conventional tablets, have drawn increasing attention recently [17,18].

With polyvinylpyrrolidone (PVP) as the filament-forming polymer matrix and ibuprofen as a model poorly water-soluble drug, Yu *et al*. firstly reported the preparation of oral fast disintegrating non-woven mats using a single fluid electrospinning process; the mats were able to release the contained ibuprofen in several seconds [5]. However, the exploitation of electrospinning in preparing FDDS is at present still somewhat limited in that almost all the reported electrospun FDDS are produced by single fluid electrospinning with a guest active ingredient distributed in the host polymer [5,19,20]. When there is no suitable solvent for synchronously meeting the two criteria, *i.e.*, having good solubility of the active ingredient and endowing the polymer’s fine electrospinnability, the preparation of FDDS using single fluid electrospinning would be a failure.

Over the past few years, electrospinning technology has evolved from using single, coaxial and side-by-side electrospinning, to adopting multiple fluids systems. These techniques allow the formation of new types of sophisticated nanofibres with well-defined microstructures, novel morphologies and/or new functions [19–21]. Particularly, coaxial electrospinning, in which a concentric spinneret can accommodate two different liquids, expands the capability of single fluid electrospinning in the preparation of nanofibres. It has been reported to prepare nanofibres from materials that lack filament-forming properties and enclosing functional liquids within the fibre matrix [22,23]. Thus, coaxial electrospinning should provide new tools for the preparation of new FDDS.

Based on above-mentioned knowledge, this study aimed to prepare FDDS of a poorly water-soluble drug quercetin using coaxial electrospinning. Quercetin is a plant pigment (flavonoid) found in many plants and foods. It is used for treating conditions of the heart and blood vessels, high cholesterol, heart disease, diabetes, for preventing cancer, for treating chronic infections of the prostate and for increasing endurance and improving athletic performance [24,25]. Fast dissolving and onset of action for the patients’ convenience and a more effective therapeutic effect are desired. To the best of our knowledge, this is the first report about fast disintegrating quercetin-loaded drug delivery systems fabricated using coaxial electrospinning.

## 2. Results and Discussion

### 2.1. Coaxial Electrospinning

**Figure 1:**
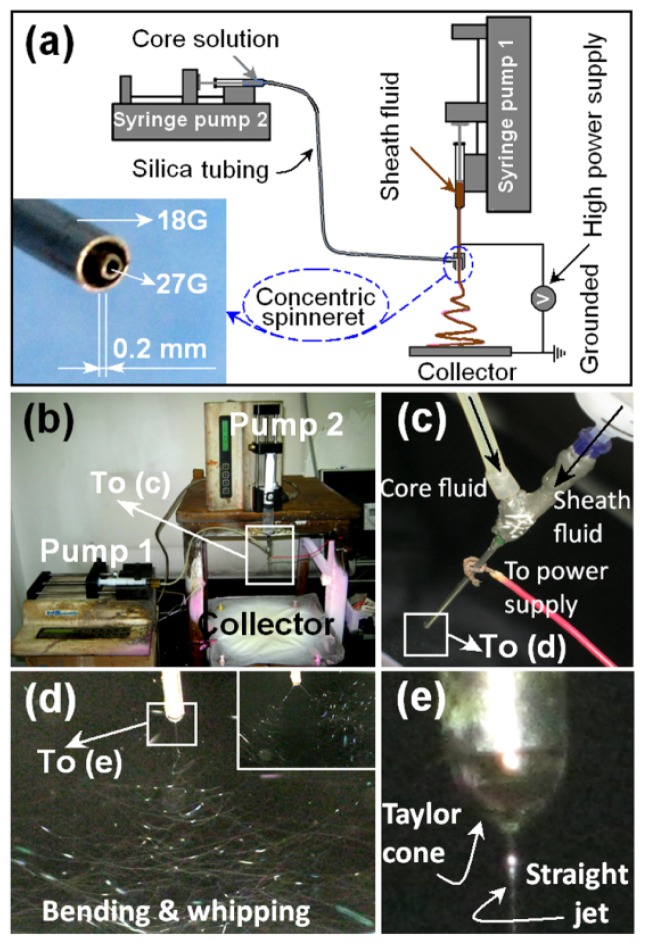
Coaxial electrospinning: (**a**) Schematic diagram of the process; the inset shows the nozzle of the homemade concentric spinneret; (**b**) Digital image showing the apparatus arrangement; (**c**) Connection between the power supply and the spinneret; (**d**) A typical fluid jet trajectory; the inset division of fluid jets is a result of a higher applied voltage of 16 kV; (**e**) A typical compound Taylor cone of the core/sheath fluids. Under a voltage of 14 kV and a collected distance of 20 cm with the flow rate of the sheath and core fluids are 1 and 0.7 mL/h, respectively, for the preparation of nanofibre F3.

A schematic diagram of the coaxial electrospinning process is shown in Figure 1a; its inset shows a digital picture of the homemade concentric spinneret, which was prepared simply by inserting a small stainless steel tube (27G; the outer and inner diameters are 1.25 and 0.84, respectively) into a big stainless steel tube (18G; the outer and inner diameters are 0.42 and 0.21, respectively). The inner tube projected out from the outer tube by 0.2 mm to facilitate easier envelopment of the core solution by the sheath fluid. The digital images of the apparatus’ arrangement and connection between the power supply and the spinneret are shown in Figure 1b,c. Under the selected conditions of electrospinning for preparing core-sheath nanofibres, the coaxial electrospinning processes were conducted smoothly and conditionally; a typical fluid jet trajectory and a typical compound Taylor cone of the core/sheath fluids are shown in Figure 1d,e. Three types of nanofibres were successfully fabricated, and some key parameters are listed in Table 1.

Quercetin has poor solubility at ambient temperature, not only in water, but also in common organic solvents, such as ethanol, methanol, acetone and chloroform. Quercetin is soluble in *N*,*N*-dimethylacetamide (DMAc); however, DMAc is not a good solvent for preparing an electrospinnable PVP solution, because of its high boiling point (166 °C). For coaxial electrospinning, the core solution does not need to have electrospinnability, and the sheath solution acts as a guide and surrounds the core liquid. The sheath solution is critical, and the sheath polymer-solvent system selected should be electrospinnable by itself to facilitate the formation of a core-sheath structure in the nanofibres [26]. Thus, although the core solution consisted of 10% (*w*/*v*) PVP and 4% (*w*/*v*) quercetin in a mixed solvent of DMAc, ethanol (3:7, *v:v*) was un-electrospinnable; however, the electrospinnable sheath fluid consisting of 10% (*w*/*v*) PVP, 0.2% (*w*/*v*) sodium dodecyl sulphate (SDS) in a 95% (*v*/*v*) ethanol aqueous solution was able to ensure a smooth coaxial electrospinning process and the formation of core-sheath nanofibres.

The coaxial electrospinning process could be changed to the single fluid electrospinning process by adjusting the flow rate of one of the fluids to 0 mL/h. When the core fluid flow rate was adjusted to 0 mL/h, the nanofibres, F1, were successfully generated. When the sheath fluid flow rate was adjusted to 0 mL/h, solid nanofibres from core solutions cannot be prepared, due to the high boiling point of DMAc. When a higher core-to-sheath fluid flow rate ratio of 1:1 was taken (0.5 mL/h to 0.5 mL/h), the electrospinning process was very unstable, the sheath fluid often penetrated the core fluid to destroy the collected nanofibre mats. Additionally, higher applied voltages would result in frequent division of the concentric fluid jets, which is disadvantageous for the uniform structure of core-sheath nanofibres. The inset of Figure 1d shows a typical division of the straight fluid jet under an applied voltage of 16 kV.

### 2.2. Morphology and Structure of Nanofibres

**Figure 2:**
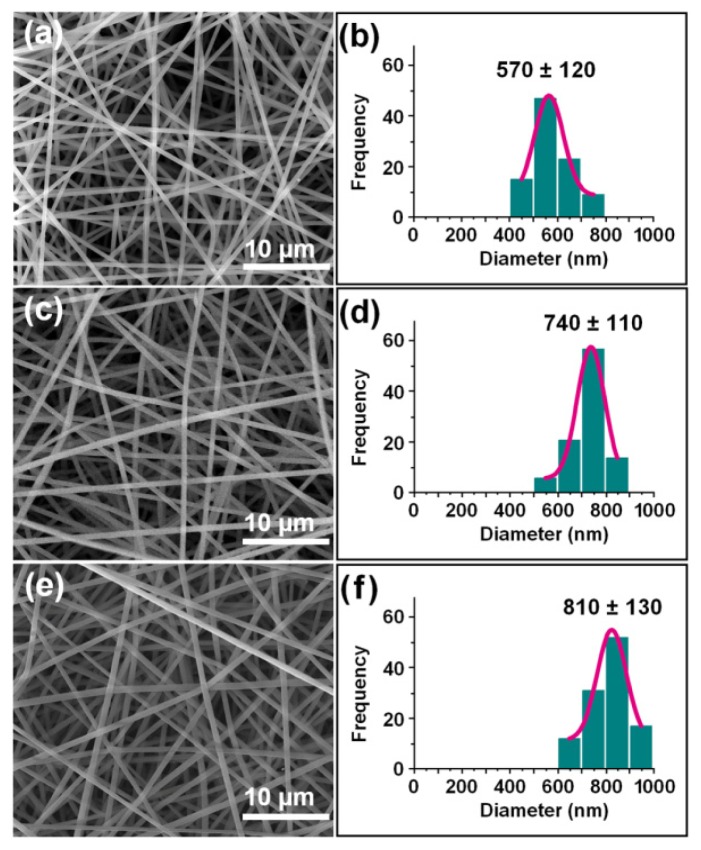
Field emission scanning electron microscope (FESEM) images of the electrospun nanofibres and their diameter distributions: (**a** and **b**) F1; (**c** and **d**) F2; (**e** and **f**) F3.

As shown in Figure 2, all the three types of nanofibres had smooth surfaces and uniform structures without any beads-on-a-string morphology. No drug particles appeared on the surface of the fibres, suggesting good compatibility between the polymers and quercetin. The nanofibres, F1, prepared through single fluid electrospinning had average diameters of 570 nm ± 120 nm (Table 1; Figure 2a,b). The core/sheath nanofibres, F2 and F3, had average diameters of 740 nm ± 110 nm (Table 1; Figure 2c,d) and 740 nm ± 110 nm (Table 1; Figure 2e,f), respectively.

**Figure 3:**
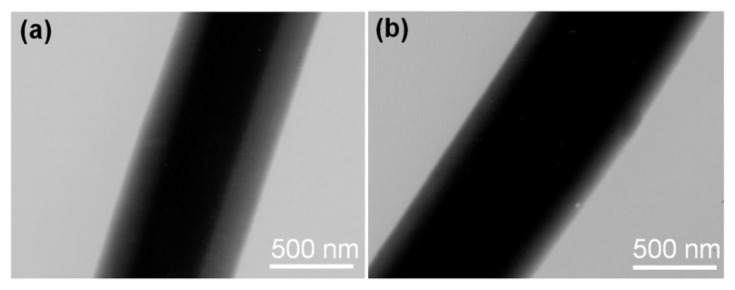
TEM images of the core/sheath nanocomposites: (**a**) F2 and (**b**) F3.

The nanofibres, F2 and F3, had clear core/sheath structures, with an estimated sheath thickness and core diameter of 400 nm and 180 nm for F2 and a value of 600 nm and 100 nm for F3 (Figure 3). Similar to the field emission scanning electron microscope (FESEM) results, no nanoparticles were discerned in the sheath and core parts. This finding suggests that these nanofibres have a homogeneous structure. The fast drying electrospinning process not only propagated the physical state of the components in the liquid solutions into the solid nanofibres, but also duplicated the concentric structure of the spinneret on a macroscale to nanoproducts on a nanoscale. As a result, the components in the sheath and core fluids occurred in the sheath and core parts of the nanofibres, respectively, with weak diffusion. Just as anticipated, the nanofibres of F3 (Figure 3b) had larger diameters and thicker sheath parts than those of F2 (Figure 3a). This difference could be attributed to the larger core flow rate for preparing F3 than for F2.

### 2.3. Physical Status and Compatibility of Components

**Figure 4:**
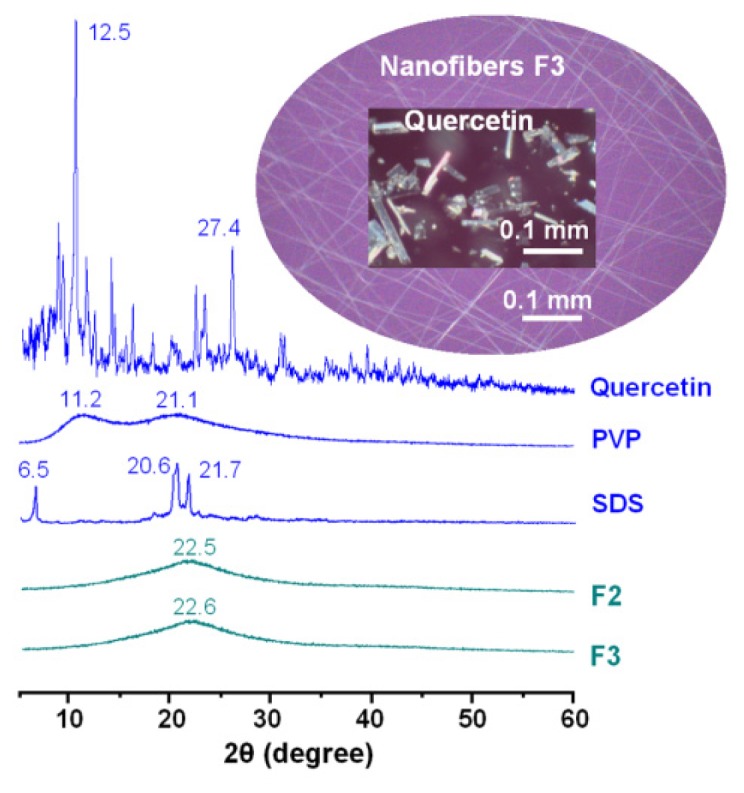
Physical status characterization: X-ray diffraction (XRD) patterns of the raw materials (quercetin, PVP and SDS) and the core-sheath nanofibres: F2 and F3 prepared by coaxial electrospinning.

Differential scanning calorimetry (DSC) and X-ray diffraction (XRD) analyses were conducted to determine the physical state of quercetin in the core-sheath nanofibres. Quercetin, a yellowish green powder to the naked eye, comprises polychromatic crystals in the form of prisms or needles. The quercetin crystals are chromatic and exhibit a rough surface under cross-polarized light, while in sharp contrast, the core-sheath nanofibres show no colour (the inset of Figure 4). The data in Figure 4 show the presence of numerous distinct reflections in the XRD pattern of pure quercetin, similarly demonstrating its existence as a crystalline material. The raw SDS is a crystalline materials, suggested by the several distinct reflections. The PVP diffraction patterns exhibit a diffuse background with two diffraction haloes, showing that the polymers are amorphous. The patterns of fibres F2 and F3 showed no characteristic reflections of quercetin, instead consisting of diffuse haloes. Hence, the core-sheath nanofibres are amorphous: quercetin is no longer present as a crystalline material, but is converted into an amorphous state in the fibres.

**Figure 5:**
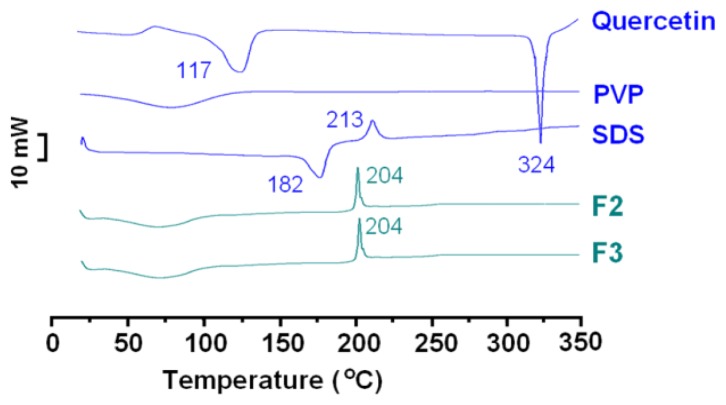
Physical status characterization: differential scanning calorimetry (DSC) thermograms of the raw materials (quercetin, PVP and SDS) and the core-sheath nanofibres, F2 and F3, prepared by coaxial electrospinning.

DSC thermograms are shown in Figure 5. The DSC curve of pure quercetin exhibits two endothermic responses corresponding to its dehydration temperature (117 °C) and melting point (324 °C), followed by rapid decomposition. SDS had a melting point of 182 °C, followed closely by a decomposing temperature of 213 °C. Being an amorphous polymer, PVP does not show fusion peaks. DSC thermograms of the core-sheath nanofibres, F2 and F3, did not show the characteristic melt of quercetin, suggesting that the drug was amorphous in the nanofibre systems. On the other hand, the decomposition bands of SDS in the composite nanofibres were narrower and higher than that of pure SDS, reflecting that the SDS decomposition rates in nanofibres are bigger than that of pure SDS. The peak temperatures of decomposition shifted from 204 °C for the nanofibres, reflecting that the onset of SDS decomposition in nanofibres is earlier than that of pure SDS. The amorphous state of SDS and highly even distributions of SDS in nanofibres should make SDS molecules respond to the heat more sensitively than pure SDS particles, and the nanofibres might have better thermal conductivity than pure SDS. Their combined effects prompted the SDS in nanofibres to decompose earlier and quicker. The DSC and XRD results concur with the SEM and TEM observations, confirming that the core-sheath fibres were essentially structural nanocomposites.

**Figure 6:**
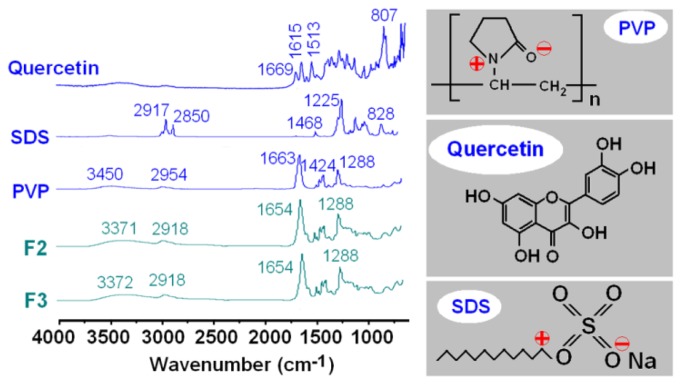
Compatibility investigation: attenuated total reflectance-Fourier transform infrared (ATR-FTIR) spectra of the components (quercetin, PVP and SDS) and their electrospun core-sheath nanofibres, F2 and F3.

Attenuated total reflectance-Fourier transform infrared (ATR-FTIR) analysis was conducted to investigate the compatibility among the electrospun components. Quercetin PVP molecules possess free hydroxyl groups (potential proton donors for hydrogen bonding) and/or carbonyl groups (potential proton receptors; see Figure 6). Therefore, hydrogen bonding interactions between quercetin can occur within the core parts of nanofibre F2 and F3. ATR-FTIR spectra of the components and their nanofibres are shown in Figure 6. Three well-defined peaks are visible for pure crystalline quercetin, at 1669, 1615 and 1513 cm^−1^ corresponding to its benzene ring and –C=O group. All three peaks disappear after quercetin is incorporated into the core of nanofibres F2 and F3, and they are merged into a single peak at 1654 cm^−1^ in them. Almost all peaks in the fingerprint regions of quercetin have shifted, decreased in intensity or totally disappeared in the nanofibres’ spectra, which suggests that hydrogen bonding occurs between quercetin and PVP. In the sheath parts of nanofibres F2 and F3, the SDS molecules could distribute in the PVP matrix, due to the electrostatic interactions between the negatively charged SDS head group, the nitrogen atom on the pyrrolidone ring of PVP [27] and, also, the attractive interaction between the negatively charged PVP oxygen (N^+^ = C–O^−^) and the electron poor C-1′ of SDS [28].

### 2.4. Fast Disintegrating Properties

Since quercetin has a UV absorbance peak at λ_max_ = 371 nm, the amount of quercetin released from the fibres is easily determined by UV spectroscopy using a predetermined calibration curve: *C* = 15.95*A* − 0.0017 (*R^2^* = 0.9997), where *C* is the quercetin concentration (μg mL^−1^) and *A* is the solution absorbance at 371 nm (linear range: 2 μg mL^−1^ to 20 μg mL^−1^). The observed content of quercetin in all the fibres was equivalent to the calculated value, suggesting no drug loss during the electrospinning process.

**Figure 7:**
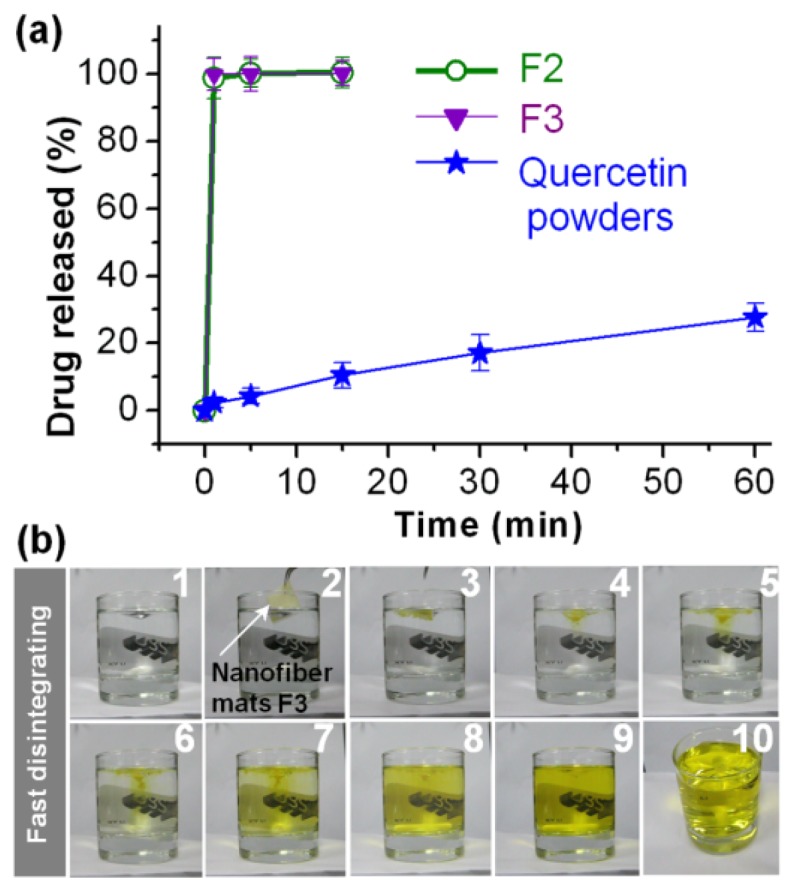
*In vitro* dissolution tests: (**a**) *In vitro* drug release profiles of the quercetin-loaded nanocomposites; (**b**) Photographs of the disintegrating process of nanofibres F3. The fast-dissolving process is shown in sequence from 1 to 10.

The nanofibres of F2 and F3 disappeared instantly after they were placed in the dissolution media. The *in vitro* drug release profiles of the core-sheath nanofibres, F2 and F3, are shown in Figure 7a, verifying that quercetin was dissolved completely into the bulk media in one minute and suggesting that they are good oral fast-disintegrating drug delivery systems. A more intuitionistic observation of the fast dissolution process is exhibited in Figure 7b: a sheet of nanofibres F3 with a weight of 40 mg was put into 200 mL physiological saline (PS) solution, and the process was recorded using video. Photographs of the disintegrating process of nanofibres F3 are shown. The fast release of quercetin from the core-sheath nanofibres F3 shown in sequence from one to 10 happened in 20 min. The yellow colour changes of the bulk solutions clearly reflected the dissolution process of quercetin, *i.e.*, the disintegrating of nanofibre mats, the release of quercetin from the nanofibres and the diffusion of quercetin from a locality to the whole bulk solution until the whole bulk solution homogeneously showed a yellow colour.

The reasons for this can be concluded as follows. First, PVP has hygroscopic and hydrophilic properties, and polymer-solvent interactions are stronger than polymer-polymer attraction forces. Thus, the polymer chain can absorb solvent molecules rapidly, increasing the volume of the polymer matrix and allowing the polymer chains to loosen out from their coiled shape. Second, the three-dimensional continuous web structure of the membrane can offer a huge surface area for PVP to absorb water molecules, greater porosity for the water molecules to diffuse into the inner part of the membrane and void space for the polymer to be swollen and disentangled and for the dissolved quercetin molecules to disperse into the bulk dissolution medium. Third, the drug and the matrix polymer formed composites at the molecular level. Fourth, SDS, as a surfactant, not only facilitates the electrospinning process through reducing the surface tension of the sheath fluids, but also enhances the hydrophilicity and wettability of the core-sheath nanofibres and, thus, promotes their fast disintegrating processes to release the contained quercetin. The synergistic actions of the above-mentioned factors should make quercetin molecules dissolve almost simultaneously with PVP molecules. That is, the capability of these nanofibres to improve significantly the dissolution rate of poorly water-soluble drugs is attributable to the reasonable selections of drug carriers, the unique properties of the nanosized fibres, the web structure of the mats and the amorphous drug status in the filament-forming matrix.

## 3. Experimental Section

### 3.1. Materials

Quercetin (purity >98%, No. MUST-12072505) was purchased from the Beijing Aoke Biological Technology Co. Ltd. (Beijing, China). PVP K60 ( $\bar{M_{w}}=360,000$) was purchased from Shanghai Yunhong Pharmaceutical Aids and Technology Co., Ltd. (Shanghai, China). Sodium dodecyl sulphate (SDS), *N*,*N*-dimethylacetamide (DMAc) and anhydrous ethanol were purchased from Sinopharm Chemical Reagent Co., Ltd. (Shanghai, China). All other chemicals used were of analytical grade, and water was doubly distilled before use.

### 3.2. Electrospinning

The core solutions were prepared by dissolving 10 g PVP and 4 g quercetin in a 100 mL mixture of ethanol and DMAc with a volume ratio of 7:3. The sheath solution was prepared by placing 10 g PVP and 0.2 g SDS in 95% (*v*/*v*) aqueous solution. Two syringe pumps (KDS100 and KDS200, Cole-Parmer, IL, USA) and a high-voltage power supply (ZGF 60kV/2 mA, Shanghai Sute Corp., Shanghai, China) were used for coaxial electrospinning. All electrospinning processes were carried out under ambient conditions (22 °C ± 3 °C, with a relative humidity of 62% ± 5%). A homemade concentric spinneret was used to conduct both single fluid (adjusting the core fluid flow rate to 0 mL/h) and coaxial electrospinning processes. A silica tubing (outer and inner diameters of 4 and 2 mm, respectively) was exploited to connect the entrance of the concentric spinneret with the syringe containing the core fluid (Figure 1a–c). The electrospinning process was recorded using a digital video recorder (PowerShot A490, Canon, Tokyo, Japan). For optimization, the applied voltage was fixed at 14 kV, and the fibres were collected on aluminium foil at a distance of 20 cm.

### 3.3. Characterization

#### 3.3.1. Morphology

The morphology of the fibre mats was assessed using an S-4800 field emission scanning electron microscope (FESEM, Hitachi, Tokyo, Japan). Prior to the examination, the samples were platinum sputter-coated under a nitrogen atmosphere to render them electrically conductive. Images were recorded at an excitation voltage of 10 kV. The average fibre diameter was determined by measuring their diameters in FESEM images at more than 100 places using the NIH Image J software (National Institutes of Health, MD, USA). Transmission electron microscope (TEM) images of the samples were recorded on a JEM 2100F field emission TEM (JEOL, Tokyo, Japan). TEM samples of the core/sheath nanofibres were collected by fixing a lacy carbon-coated copper grid on the collector. The topographies of the raw quercetin particles and the nanofibres, F3, were observed under cross-polarized light using an XP-700 polarized optical microscope (Shanghai Changfang Optical Instrument Co., Ltd., Shanghai, China).

#### 3.3.2. Physical Status and Compatibility

The X-ray diffraction analysis (XRD) was conducted using a D/Max-BR diffractometer (RigaKu, Japan) with Cu Kα radiation in a 2θ range of 5° to 60° at 40 mV and 300 mA. Differential scanning calorimetry (DSC) was carried out using an MDSC 2910 differential scanning calorimeter (TA Instruments Co., New Castle, DE, USA). Sealed samples were heated at 10 °C/min from 20 to 350 °C. The nitrogen gas flow rate was 40 mL/min. Attenuated total reflectance-Fourier transform infrared (ATR-FTIR) spectroscopy was carried out on a Nicolet-Nexus 670 FTIR spectrometer (Nicolet Instrument Corporation, Madison, WI, USA) at a range of 500 cm^−1^ to 4000 cm^−1^ and a resolution of 2 cm^−1^.

#### 3.3.3. *In Vitro* Dissolution Tests

*In vitro* dissolution tests were carried out according to the Chinese Pharmacopoeia, Method II, a paddle method, was performed using a RCZ-8A dissolution apparatus (Tianjin University Radio Factory, Tianjin, China). An equal amount of quercetin (*i.e.*, 30 mg raw powder, 263 mg nanofibres F2 and 182 mg nanofibres F3) were placed in 900 mL of physiological saline (PS, 0.9 wt%) at 37 ± 1 °C. The instrument was set to stir at 50 rpm, providing sink conditions with *C* < 0.2*C*_s_. At predetermined time points, 5.0-mL aliquots were withdrawn from the dissolution medium and replaced with fresh medium to maintain a constant volume. After filtration through a 0.22 μm membrane (Millipore, MA, USA) and appropriate dilution with PS, the samples were analysed at λ_max_ = 371 nm using a UV-vis spectrophotometer (UV-2102PC, Unico Instrument Co. Ltd., Shanghai, China). The cumulative amount of quercetin released was back-calculated from the data obtained against a predetermined calibration curve. The experiments were carried out six times, and the accumulative percent reported as mean values was plotted as a function of time (*T*, min).

## 4. Conclusions

Fast disintegrating quercetin-loaded drug delivery systems in the form of non-woven mats were successfully fabricated using coaxial electrospinning. The drug contents in the nanofibres can be manipulated through adjusting the core-to-sheath flow rate ratio. FESEM images demonstrated that the nanofibres prepared from the single sheath fluid and double core/sheath fluids (with core-to-sheath flow rate ratios of 0.4 and 0.7) have linear morphology with a uniform structure and smooth surface. The TEM images demonstrated that the fabricated nanofibres had a clear core-sheath structure. DSC and XRD results verified that quercetin and SDS were well distributed in the PVP matrix in an amorphous state, due to the favourite second-order interactions. *In vitro* dissolution experiments verified that the core-sheath composite nanofibre mats could disintegrate rapidly to release quercetin within one minute. The study reported here provides an example of the systematic design, preparation, characterization and application of a new type of structural nanocomposite as a drug delivery system for fast delivery of poor water-soluble drugs.

## Figures and Tables

**Table 1:** Parameters used for electrospinning and details of the fibre products.

No.	Process	Flow rate of fluid (mL/h)		Drug content in products	Morphology c	Diameter (nm)

		Sheath a	Core b			
F1	Single	1.0	—	0	Linear	570 ± 120
F2	Coaxial	1.0	0.4	11.4%	Linear	740 ± 110
F3		1.0	0.7	16.5%	Linear	810 ± 130

a The sheath fluid consists of 10% (*w*/*v*) polyvinylpyrrolidone (PVP) and 0.5% (*w*/*v*) of sodium dodecyl sulphate (SDS) in 95% (*v*/*v*) ethanol aqueous solutions;

b The core fluid consists of 10% (*w*/*v*) PVP and 4% (*w*/*v*) of quercetin in a mixture of ethanol and *N*,*N*-dimethylacetamide (DMAc) (7:3 *v*/*v*);

c “Linear” morphology refers to nanofibres with few beads or spindles.
